# Special Materials Used for Casts in Vulcanite Work

**Published:** 1915-05

**Authors:** J. H. Prothero


### SPECIAL MATERIALS USED FOR CASTS IN VULCANITE WORK.

J. H. PROTHERO, D.D.S.

It is a well-established fact that when plaster is subjected to stress, or compressive force, the surface crystals, or those first taking the load, crush and break down, when the stress passes the modulus of resistance of the material.

It has also been demonstrated, that the force ordinarily exerted in closing an overpacked flask is far in excess of that which plaster can stand without the crystals crushing and the face of the cast becoming distorted.

The effect of such distortion, be it small or great, on casts against which vulcanite or celluloid dentures are molded, is to impair, if not altogether destroy, the adaptation of the dentures to the oral tissues.

Two ways are possible for lessening if not entirely overcoming the danger of distortion of dentures, due to yielding of the plaster casts under the influence of excessive pressure, heat, and moisture, during vulcanization.

The first of these does not require the use of any special materials other than those commonly employed in the dental laboratory. The method of technic differs in some respects from that usually followed in such procedures, the variations being noted under the *closing of flasks*, preparatory to vulcanizing.

The second plan requires that the cast be constructed of a material harder and more resistant to stress, and to deleterious influences in general than is plaster.

Several materials, capable of withstanding much greater crushing strain than plaster, may be made use of, the properties of the three most important of which will now be considered in the order of their value, viz., *oxychloride of magnesium*, *Spence's plaster*, and *tin*.

All of the artificial stone of the finer quality may be classed among the silicates or oxychlorides. Pozzuolana is an exception to this, as the French product consists of the oxides of seven elements and water. The oxides of silicon, aluminum, iron and calcium furnish ninety per cent. of the bulk, while magnesium, potassium, sodium, and water make up the remainder.

Through data furnished by the late Prof. Vernon J. Hall, some experiments were made with the oxide and chloride of magnesium. The results, however, were not encouraging for reasons not then apparent, but which were later disclosed. To illustrate: in building operations, the *commercial* oxide and chloride are combined with sawdust for flooring purposes, for two reasons, first, because the commercial oxide is much less expensive than the chemically pure material, and second, because the latter will not produce as hard or permanent a substance when set as the commercial variety.

Since the commercial products work satisfactorily for flooring and in other industrial lines, this variety was used in the experiments referred to. From the first, difficulties were encountered, due principally to cracking of the material in setting, and to a very perceptible contraction in the mass.

Cracking was later found to be due to the presence of carbondioxide in the oxide of magnesia. Its presence may be accounted for in two ways. Magnesium oxide is produced usually by burning magnesium carbonate, just as calcium oxide or lime is produced by burning calcium carbonate. In both cases the carbondioxide is driven off when the process is properly conducted. Unless the calcinaction is thorough all of the $CO_2$ will not be eliminated. Again, the oxide of magnesia may be properly prepared, but if left exposed to the air, it will take up moisture and carbondioxide, and gradually return to the carbonate. From whatever source it may come, its presence in the oxide of magnesia renders the latter worthless for cast construction. The remedy consists in recalcining the mixed oxide and carbonate above a red heat, to expel the $CO_2$, or if this is not practicable, discard it for a better grade of material.

Contraction in the hardened mass, noticeable in the space seen between the impression and cast when the latter has hardened, is the result of using an under-saturated solution of the chloride, the liquid with which the powdered oxide is combined. By increasing the strength of the liquid to full saturation, adding crystal chloride until there is a slight excess in the bottom of the stock vessel, contraction can be overcome.

The length of time required for setting—about twelve hours—is considered an objection by some, but the advantages gained in more perfect adaptation of the denture to the oral tissues and in increased density of the vulcanite far outweigh the disadvantages mentioned.

### OXYCHLORIDE OF MAGNESIA—ADVANTAGES.

The principal advantages of oxychloride of magnesium for casts in vulcanite work are these: hardness, density, smoothness of surface and an extremely low expansive index, less than one-fourth that of the best grades of plaster. It is sufficiently impervious to moisture and heat to maintain its form without crushing, even under heavy pressure. The writer has vulcanized two baseplates on the same cast, both of which showed satisfactory adaptation to the oral tissues. At the end of the second vulcanization, the cast, although permeated with moisture, was quite hard and resistant to stress and on evaporation of the moisture appeared much harder than casts constructed from the best grades of plasters before vulcanization.

Rubber of any shade vulcanized in contact with oxychloride of magnesium is hard, dense, elastic, capable of taking a high polish, and on account of the density of the cast is practically free from nodules.

Partial dentures vulcanized on casts of this material show all the characteristic lines and fine surface markings of the teeth and tissues against which it is molded as clearly as an accurate plaster impression can reproduce them, because the cast is not changed or defaced in the slightest degree by manipulative procedures.

In tests made for expansion, the greatest movement registered, from the buccal face of one tuberosity to the corresponding opposite surface, was 15-10,000 of an inch, against from 60-10,000 to 100-10,000 of an inch, in casts made from the best grades of plaster.

Comparative compression tests on blocks of plaster and of magnesium compound set over night, showed the following result: size of blocks 1½x1½x½ inches; area of plunger, ¼ inch. In the plaster blocks, the plunger began sinking into the block at twenty pounds, and under continued pressure penetrated about ⅛ inch, the block breaking at 100 pounds.

The magnesium blocks showed no perceptible compression up to 1,000 pounds, at which point they suddenly crushed. In one-inch cubes, the oxychloride of magnesium will stand a crushing strain of nearly 5,000 pounds, according to Major Gilmore, U. S. A.

### MATERIALS USED FOR CAST CONSTRUCTION.

The following instruction in reference to procuring and handling the magnesium materials for casts covers the essential points to be kept in mind.

### MAGNESIUM OXIDE.

There are two varieties of magnesium oxide, known as the *light* and *heavy* oxide. The difference, which is one of specific gravity and not of chemical constitution, is brought about by the manner in which the magnesite ($MgCO_3$) is burned. In "Cements, Limes, and Plasters," E. C. Eckel says: "If $MgCO_3$ be strongly heated, the effect, as with lime carbonate, is to drive off the $CO_2$, leaving the $MgO$ as a white solid. A curious and technologically important phenomenon connected with the temperature employed is to be noted. If the calcination is carried on quickly at a red heat the magnesia resulting will have a specific gravity of 3.00 to 3.07, while if the calcination is long continued or carried on at a higher temperature the resulting MgO will be much denser, possessing a specific gravity of 3.61 to 3.80.''

For the construction of casts, the heavy oxide commercially known as *powdered magnesite,* or *calcined magnesia,* should be employed. To prevent its return to $MgCO_3$, by absorbing $CO_2$ and moisture from the air, as indicated by its becoming granular and lumpy, it should be kept in air-tight containers, just as plaster must be protected in damp climates.

### MAGNESIUM CHLORIDE.

Magnesium chloride is a crystalline, deliquescent substance, having much the appearance of sea salt. It is obtained in several ways, a common source being by heating magnesium ammonium chloride ($MgCl_2$ $NH_4$ Cl) to about 460 C. The ammonium chloride volatilizes, leaving anhydrous $MgCl_2$.

The ordinary commercial product, instead of the chemically pure chloride, is suitable for use in cast construction; some of the commercial products occasionally contain $HSO_4$ as an impurity. When present, in the chloride solution, it will in time render the hardened mass of oxychloride somewhat soluble in water. The sulphuric acid can be eliminated by adding barium hydrate to the chloride solution. When the resulting precipitate barium sulphate ceases to form, it indicates that the acid has been neutralized. From six per cent. to ten per cent. by weight of the reagent compared with the chloride is required. This method of neutralizing the acidity of the magnesium chloride solution is more strongly indicated when the oxychloride material is to be wrought into work of a permanent character and is not as essential for vulcanite casts, since the latter are destroyed after vulcanization, in removal from the dentures. After two years almost constant use of magnesium oxychloride, the writer has not found it necessary to purify any commercial chloride solution, but has frequently been obliged to recalcine the oxide to drive off the $CO_2$, as previously mentioned.

PREPARING THE MAGNESIUM CHLORIDE SOLUTION.

In the average dental practice but a comparatively small amount of magnesium oxychloride will be used in the course of a month, so the preparation of a large quantity is not advisable. It is a better plan to make up one or two quarts of the liquid chloride, renewing the solution from time to time as needed. In this way the quality of the liquid can easily be kept up to standard.

To make the solution, put two pints of water in a clean, two-quart glass bottle, and add the crystal chloride until complete saturation of the water is effected. The visible test of full saturation of the water appears in the presence of undissolved crystals of chloride in the bottom of the vessel.

A half-inch layer of crystals in the bottom of the vessel at all times will do no harm and will keep the solution fully saturated. If the crystals disappear, add more until the usual amount of excess is restored and if they increase, due to evaporation of the water, add more of the latter. After full saturation of the water has occurred, the liquid should not be shaken up nor the crystals disturbed in decanting off a portion for use.

MANNER OF MIXING THE OXIDE WITH THE CHLORIDE SOLUTION.

Place a sufficient amount of the chloride solution to form a cast in the plaster bowl and sift in the oxide just as plaster is manipulated, stirring much more vigorously and for a longer time than when plaster is used. Additions of the oxide are made from time to time and the stirring continued until the mass is sufficiently thick to stand alone. This is an extremely important requirement, for when too thin the oxychloride overflows the bounds of the impression and the cast can not be built up to proper form.

The object in vigorous stirring is to eliminate all air that may be in the powdered magnesite and to coat every granule with a film of the liquid. The tendency of all beginners in using this material is to slight the stirring and economize on the powder, with the result that although the mass hardens well and is smooth, there is an excess of the chloride present and the cast will more readily fracture under stress than when the material is thickly mixed. It is also inclined to soften more readily during vulcanization.

### TREATMENT OF IMPRESSIONS.

To get an absolutely smooth surface to a cast of this material it is necessary to have a smooth surface to the impression. This can best be secured by treating either plaster or modeling compound impressions with a varnish having a sandarac base. Gilbert's Imperial Varnish fulfils the requirements well.

In filling modeling compound impressions with plaster the surfaces are merely moistened with water to accelerate the flow of plaster over the impressed area. With oxychloride, although moisture on the surface of the impression would insure ease of introduction, its presence would eventually deteriorate the surface of the cast, rendering it softer and less resistant to stress.

Modeling compound impressions are varnished because unless so treated the compound is at times extremely difficult to remove from the oxychloride cast.

### FILLING THE IMPRESSIONS.

The impression is filled with oxychloride mixture in much the same manner as with plaster, with this exception: Since the face of the impression is dry, for reasons previously stated, to prevent the formation of creases in the cast where two or more additions of the mixture may meet, it is best to apply each subsequent addition to an area already covered, and by vibration let the mass last added settle down and push the margins of that already adapted over the uncovered areas of the impression.

In partial cases impressions of teeth should be filled with a small tamper to avoid the confinement of air in the matrix.

The mass of oxychloride should be built to the proper form of the cast, being careful to avoid any excess, since when set, it is very difficult to cut with a knife. By adjusting a bead of wax on the periphery of the impression to outline of the extreme outer lines of the cast and building the material to this bead much annoyance will be averted later on.

GENERAL REMARKS.

By mixing from 50 to 80 per cent. of clean sand with 50 to 20 per cent. of the oxide of magnesium a harder and much more resistant mass will result than if the oxide and chloride alone are used.

By filling the impression partially with oxychloride mixture and inserting a previously selected metal model form (Kerr's), an extremely small amount of the material will be required and the peripheral outline, as well as the depth of the cast, will be kept within minimum limits, since the oxychloride need not cover the metal form on these surfaces.

The material should not be disturbed until thoroughly hardened by attempting to remove the impression. It usually requires about twelve hours to set. Should the mass have spread out over the sides of the impression much more than is desirable it may be trimmed peripherally in four or five hours after mixing without endangering the cast.—*Dominion Dental Journal.*

